# Intensification of oxidative stress and inflammation in type 2 diabetes despite antihyperglycemic treatment

**DOI:** 10.1186/1475-2840-7-20

**Published:** 2008-06-22

**Authors:** Raymond Farah, Revital Shurtz-Swirski, Olga Lapin

**Affiliations:** 1 Departement of Internal Medicine B, Ziv Medical Center, Safed, Israel; 2 Eliachar Research Laboratory, Western Galilee Hospital, Nahariya, Israel

## Abstract

**Introduction:**

The metabolic deregulation associated with diabetes mellitus (DM) causes secondary pathophysiologic changes in multiple organ systems. Endothelial injury is induced by oxidative stress (OS) and inflammation. We have previously shown that DM type 2 patients are exposed to increased OS and inflammation contributed in part by primed peripheral polymorphonuclear leukocytes (PMNLs).

**Aims:**

To characterize the effect of oral medication on PMNL priming, on PMNL-related and on systemic inflammation, in correlation to changed diabetes parameters in patient with newly diagnosed type 2 DM.

**Methods:**

PMNLs were separated from DM patient's prior and following treatment with either metformin (Glucophage), or Thiazolidinedione (rosiglitazone) and from healthy control subjects (HC). Rate of superoxide release from phorbol ester-stimulated PMNLs and CD11b on PMNLs assessed PMNL priming. White blood cells (WBC) and PMNL counts and apoptosis reflected PMNL-related inflammation. CRP, fibrinogen, transferrin and albumin blood levels reflected systemic inflammation.

**Results:**

Both metformin and rosiglitazone treatments reduced significantly the high levels of glucose and HbA1c, and slightly improved lipid profile during 2 months. PMNL priming parameters, higher compared to HC, increased after 2 months of metformin treatment. Rosiglitazone treatment decreased PMNL priming. ALP, higher in DM, significantly decreased following 2 months of both treatments. Systemic inflammation markers (fibrinogen, CRP), higher in DM, decreased following both treatments. Transferrin and albumin were similar to HC. PMNL-related inflammation markers were higher in DM; however, only PMNL apoptosis decreased after both treatments. Monocyte counts, higher in DM compared to HC, decreased following both treatments. Serum insulin levels, higher in DM compared to HC, decreased following both treatments. PMNL-related priming and inflammation parameters positively correlated with HbA1c.

**Conclusion:**

The present research adds new facet in evaluating anti-hyperglycemic treatment in type 2 DM patients. Despite sufficient glycemic control using both treatments, some PMNL-related parameters deteriorated. Thus, anti hyperglycemic treatment should be favored due to its combined anti-PMNL priming and anti-inflammatory effect, in addition to its anti-hyperglycemic characteristics, according to the correlation among these parameters. Such combined treatment may reduce morbidity and mortality common in DM patients.

## Background

Atherosclerosis begins during adolescence in otherwise healthy individuals; it develops earlier in type 2 diabetic patients and frequently is the leading cause of premature mortality. Whereas there is now highly persuasive evidence that glycemic control reduces the the risk of microvascular complications in type 1 diabetes, and probabily in type 2 diabetes as well, such evidence is unavailable for macrovascular complications. There is a close linkage bettween rapidly developing atherosclerosis in diabetic patients and endothelial dysfunction and insulin resistance in diabetes mellitus [1-3]. Endothelial dysfunction appears to predate the development of diabetic angiopathy and play a key role in the pathogenesis of diabetic angiopathy in man. Insulin resistance, oxidative stress, and inflammation all play a key role and participate in the development of endothelial dysfunction [4-7]. Much evidence support the presence of insulin resistance as the fundamental pathophysiologic disturbance for the clauster of metabolic and cardiovascular disorders, known as the metabolic syndrome. Oxidative stress is believed to play a role in diabetic-induced vascular complication [8-10]. Chronic exposure to increased levels of hydroxyl radicals *in vivo *likely plays a significant role in the origine of diabetes-associated endothelial dysfunction. Diabetic endothelium produce increase in both superoxide anion radicals and hydrogen peroxide leading to enhanced intracellular production of hydroxyl radicals that could be implicated in diabetes-induced endothelial dysfunction. Chronic treatment with a known hydroxyl radical scavenger, dimethylthiourea, could prevent endothelial dysfunction in diabetes. Other indicators for the oxidative stress in diabetes the elevated levels of advanced glycation end products (AGE) that rise in parallel with the severity and complications of the disease [11,12], as we can demonstrate this during the elevation of HbA1c in diabetes [13]. We have previously shown that in other clinical situations known to be associated with endothelial dysfunction and accelerated atherosclerosis such as uremia, hypertension and type 2 diabetes, PMN are primed, contributing to OS and inflammation [14-16]. PMNLs from diabetic patients demonstrated defects in LPS-induced apoptosis. High-glucose environment may mediate these findings. The inability of diabetic neutrophils to reduce apoptosis following LPS exposure resulted in relatively increased apoptosis. This would cause decreased functional longevity of neutrophils and increased neutrophil clearance from infectious sites, possibly contributing to the increased susceptibility and severity of infections in diabetic patients [17]. Other inflammatory markers found in diabetes as elevated levels of sCD14 [18], fibrinogen and CRP that shown a significant correlation with PMNLs [19,20]. Monocytes are the principal inflammatory cells associated with the plaque formation, adherence of monocytes to the arterial wall is an early event in the development of atherosclerotic lesions. In uncontrolled diabetes with elevated HbA1c as in hyperglycemia *in vitro*, the peripheral blood monocytes are preferentially recruited to the intima early in the development of atheroma, they adhere to endothelium, migrate to the subendothelial space, and trasformed into lipid-lader foamy macrophages [21-25]. High doses of vitamin E (800 IU/day) can decrease the recruitment of monocytes to endothelim and cause decrement in oxidized LDL and the level of various plasma interleukines [22,24]. Therefore any drug that can decrease the activation of PMNLs or the monocytes, oxidative stress and inflammation should be preferable and better than drugs that treat only the hyperglycemia. Thus, in the present study we compare PMNL priming parameters in diabetic patients, treated daily with **metformin **for two months to those treated with **rosiglitazone**.

## Research design and methods

### Study population

Thirty untreated DM patients patients were included in this study: All the participants in this study were in age range of 30–65 year and nonsmokers. Patients underwent medical evaluation including opthalmoscopic examination, ECG and laboratory tests. The following exclusion criteria that were applied for the diabetic patients: significant dyslipidemia – LDL-cholesterol ≥ 130 mg%, HDL-cholesterol ≤ 35 mg%, triglycerides ≥ 400 mg%; evidence for microvascular diseases (diabetic retinopathy and diabetic nephropathy) and evidence for macrovascular disease (coronary artery disease, cerebrovascular disease and peripheral vascular disease); obese, BMI ≥ 30; Blood pressure > 130/85.

The untreated DM patients were further enrolled in a prospective longitudinal study, being followed up for 4 and 8 weeks of either **metformin **(850 mg/day) or **rosiglitazone **(4 mg/day) treatment.

A group of age- and gender-matched healthy controls (HC, n = 20) were included in the study. The inclusion of HC participants was based upon a clinical examination with laboratory confirmation. Patients and subjects gave informed consent for blood sampling approved by the institutional committee in accordance with the Helsinki declaration.

### PMNL and sera separation

Blood was drawn in the morning after an overnight fast from hyperlipidemic patients and HC for the determination of biochemical and hematological parameters and for PMNL isolation. PMNL isolation was carried out from a 20 mL heparinized blood sample as previously described [15,16,26]. The separated PMNLs (>98% pure, approximately 10^7 ^cells per isolation) were resuspended in phosphate buffered saline (PBS) containing 0.1% glucose. Sera were used for the determination of C-reactive protein (CRP), albumin and transferrin while plasma served for fibrinogen quantification.

### PMNL priming

#### a. Rate of superoxide release

The measurements of the rate of superoxide release are based on superoxide dismutase (SOD) inhibitable reduction of 80 μM cytochrome C (Sigma, St. Louis, MO., USA) to its ferrous form. The rate of superoxide release was monitored from 10^6 ^separated PMNLs at 22°C up to 90 min as previously described [15,16,27].

#### b. Membrane CD11b

The PMNL membrane adhesion molecule CD11b was determined by flow cytometry, reflecting PMNL priming [28]. Whole blood was treated with 0.1% formic acid to lyse the erythrocytes, and mouse anti human-CD11b phycoreythrin (PE)-conjugated antibody (Immunotech, Marseille, France) was added. Anti-human CD16 PC5-conjugated antibody (Immunotech, Marseille, France) was also used in all samples to enable gating on the PMNL population.

### PMNL-derived inflammation

#### a. WBC, PMNL and monocyte counts

Counts of WBC, PMNLs and monocytes from blood drawn in EDTA were performed by an automated cell counter (Coulter STKS, Miami, Fla., USA).

#### b. Analysis of apoptotic PMNLs

Apoptosis was determined in whole blood from 20 randomly chosen hyperlipidemic patients and 20 HC by flow cytometry according to Kuypers et al. [29]. Briefly, Annexin V kit (Bender MedSystems, Vienna, Austria) was applied to blood samples after lysis of red blood cells by Q prep (Beckman Coulter, Fullerton, California, USA). Monoclonal anti-CD16 labeled with PC5, was used for gating on PMNLs.

### Systemic inflammation

#### a. Positive acute phase protein

##### Fibrinogen and CRP

Fibrinogen levels were measured in plasma using the K-Assay^® ^kit (Kamiya Biomedical Company) by chemical analyzer (Cobas Mira, Roche Diagnostics, Germany). Blood CRP levels were measured using commercial kit (Biokit, Spain) by the Hitachi 917 Automatic analyzer (Roche Diagnostics, Germany).

#### b. Negative acute phase proteins

##### Albumin and transferrin

Blood albumin (Alb plus, Roche Diagnostics, Manheim, Germany) and transferrin (Transferrin ver.2, Roche Diagnostics, Manheim, Germany) levels were measured using the Hitachi 917 Automatic analyzer (Roche Diagnostics, Germany).

### Statistical Analysis

Data values are means ± SD. The two groups were compared by student t-test, using Prism version 3.0 statistical software (GraphPad software, San Diego, Ca). Correlations between different study parameters were performed using Pearson correlation coefficients. *P *< 0.05 was considered significant.

## Results

### Study population

Table 1 summarizes the biochemical characteristics of the participants. All studied groups of patients showed similar age range, blood pressure values, serum cholesterol, serum triglycerides, and liver enzymes, without showing target organ damage. Most traditional risk factors were similar during antihyperglycemic treatment period. ALP was higher in DM compared to HC and decreased during 2 months of both treatments (Table 1). Along with its role as an hepatic enzyme, former studies have shown significantly higher blood level of this enzyme in hypertensive patients and rats, following degranulation from primed PMNLs [14]. The present study adds DM patients to this finding, and shows ameliorating results following both antihyperglycemic treatment.

**Table 1:** The changes in DM patients' parameters.

**Parameters**	**HC**	**Metformin (months)**			**Rosiglitazone (months)**	
		**0**	**1**	**2**	**0**	**2**

**Fundus**	**neg**	**neg**	**neg**	**neg**	**neg**	**neg**

**MAP (mm Hg)**	87.0 ± 2.2	99 ± 3.2	98.1 ± 1.4	99.1 ± 3.6	96.1 ± 2.0	96.2 ± 0.2
**Glucose (mg/dL)**	94.0 ± 11.9	228.4 ± 15.1^b^	135.3 ± 8.3^a,b^	135.9 ± 3.2^a,b^	235.2 ± 15.8^b^	143.1 ± 14.1^a,b^
**HbA1c (μmol/L)**	5.4 ± 0.2	8.9 ± 0.3^b^	7.2 ± 0.5^a,b^	7 ± 0.1^a,b^	10.7 ± 0.6^b^	7 ± 0.3^a,b^
**Calculated GFR**	94.6 ± 2.9	99.5 ± 2.1	95.7 ± 2.0	94.6 ± 2.9	95.4 ± 4.8	90.1 ± 4.1
**Cholesterol (mg/dL)**	205.0 ± 1.7	203 ± 8.6	188.3 ± 9.9	188.3 ± 2.2	207.3 ± 9.0	195.1 ± 4.8
**Triglycerides (mg/dL)**	120.4 ± 3.2	153.2 ± 2.1	114.6 ± 26.3	129.3 ± 6.5	190.2 ± 3.5	141.4 ± 5.5
**HDL (mg/dL)**	58.6 ± 0.7	45.9 ± 2.5	44.7 ± 3.4	43.9 ± 0.9	42.04 ± 2.4	41.3 ± 0.9
**LDL (mg/dL)**	114.6 ± 1.3	128.4 ± 7.5	120.7 ± 8.7	118.6 ± 2.1	130.0 ± 7.7	114.7 ± 2.6
**Hb (g/dL)**	14.3 ± 0.1	14.6 ± 0.4	14.0 ± 0.6	14.8 ± 0.5	14.7 ± 0.3	14.4 ± 0.1
**ALT (U/L)**	20.3 ± 0.5	27.3 ± 2.8	28.4 ± 5	20.3 ± 0.5	33.5 ± 6.5	24.3 ± 0.7^a^
**AST (U/L)**	19.6 ± 0.3	19.2 ± 1.5	24 ± 3.6	19.6 ± 0.3	23 ± 3	17.4 ± 0.3^a^
**ALP (U/L)**	76.2 ± 6.1	87.3 ± 4.8^b^	73.7 ± 3.9^a^	76.2 ± 6.1^a^	83.8 ± 4.5^b^	75.3 ± 1.5^a^
**LDH (U/L)**	283.3 ± 1.9	311 ± 17	273.2 ± 10.8	283.3 ± 1.9	270.4 ± 10.8	255.8 ± 3.3
**Insulin (μU/mL)**	7.6 ± 0.8	15.2 ± 1.4^b^	13.5 ± 1.0^b^	11.1 ± 0.9^a,b^	17.5 ± 1.8^b^	9.5 ± 0.4^a,b^

^a ^*P *< 0.05, treated DM patients vs. untreated DM patients;

^b ^*P *< 0.05, untreated DM patients vs. HC subjects

The diabetes characteristics over 8 weeks period of antihyperglycemic treatment is depicted in Table 1: Blood HbA1c and glucose levels significantly decreased following both treatment. Insulin levels, significantly higher in DM compared to HC, decreased following both antihyperglycemic treatment (Table 1).

### Effect of metformin and rosiglitazone on diabetes characteristics and PMNL priming-related parameters

**Figure 1:**
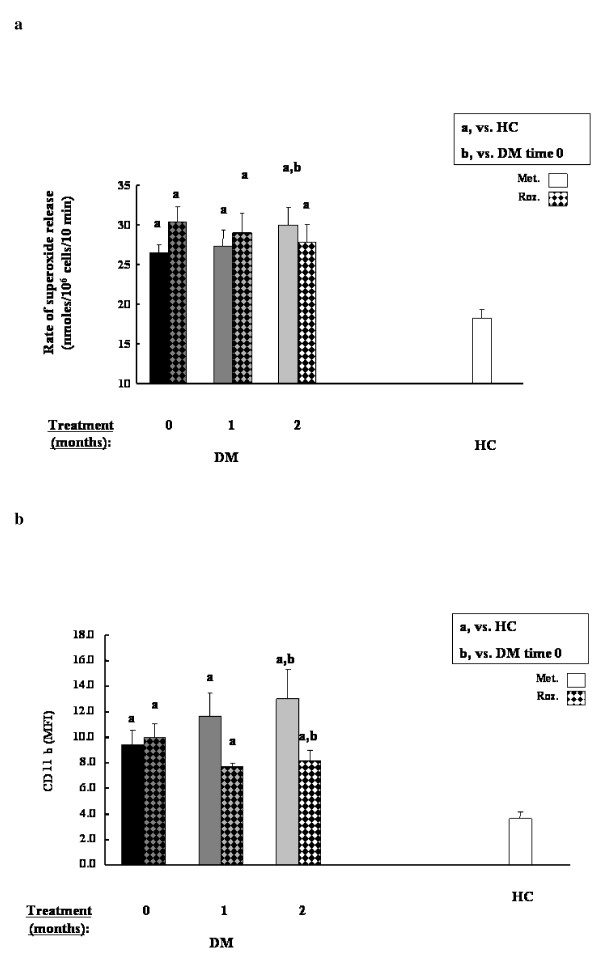
PMNL priming parameters, namely, rate of superoxide release (**a**) and PMNL CD11b levels (**b**), before and following 1 and 2 months of either metformin (***blank***) or combined Roziglytazone (***dotted***) treatment. Data are mean ± SEM. ^a^*P *< 0.05, vs. HC subjects; ^b ^*P *< 0.05, vs. untreated DM patients.

PMNL priming parameters revealed different picture with each antihyperglycemic treatment; the metformin-treated patients showed significant increase in PMNL priming, as reflected by significant increase either in the rate of superoxide release from separated PMNLs or in CD11b levels on PMNLs (Fig. 1a and 1b, respectively). However, the rosiglitazone treatment inhibited significantly either the rate of superoxide release from separated PMNLs or in CD11b levels on PMNLs (Fig. 1a and 1b, respectively). It has to be emphasized that both groups of patients showed higher values of PMNL priming compared to those measured in HC subjects.

### PMNL-derived inflammation

#### a. WBC, PMNL and monocytes counts

Counts of WBC and PMNLs, significantly higher in DM compared to HC, decreased only after rosiglitazone treatment, while metformin treatment caused a rise in these cells' count (Table 2). The significant higher level of monocytes in DM patients, decreased significantly after both antihyperglycemic treatments (Table 2).

**Table 2:** The changes in DM patients' inflammation parameters.

**Parameters**	**HC**	**Metformin (months)**			**Rosiglitazone (months)**	
		**0**	**1**	**2**	**0**	**2**

**WBC × 10^9^**	6.7 ± 0.1	7.3 ± 0.3^b^	7.4 ± 0.4	7.5 ± 0.2	7.04 ± 0.5^b^	6.8 ± 0.6
**PMNLs × 10^9^**	4.0 ± 0.1	4.6 ± 0.3^b^	4.7 ± 0.2	4.9 ± 0.1	4.2 ± 0.4^b^	4.4 ± 0.5
**PMNL apoptosis (%)**	4.0 ± 0.1	12.7 ± 3.4^b^	9.5 ± 2.8^a, b^	6.5 ± 1.0^a^	20.4 ± 4.4^b^	7.5 ± 1.9^a^
**Monocytes × 10^9^**	0.36 ± 0.02	0.44 ± 0.05^b^	0.40 ± 0.04^b^	0.39 ± 0.04^a^	0.49 ± 0.03^b^	0.39 ± 0.01^a^
**Fibrinogen (mg/dl)**	304.6 ± 17.6	440.1 ± 25.2^b^	471.1 ± 28.8^b^	415.9 ± 41.2^a, b^	400 ± 16.6^b^	332.8 ± 8.1^a,b^
**Albumin (g/dl)**	4.6 ± 0.05	4.53 ± 0.1	4.74 ± 0.1	4.7 ± 0.04	4.5 ± 0.1	4.6 ± 0.1
**Transferrin (g/dl)**	273.2 ± 10.6	274.6 ± 8.4	276.7 ± 7.2	287.9 ± 7.7	264.7 ± 7.6	286.2 ± 8.9
**CRP (mg/L)**	1.5 ± 0.1	8.3 ± 1.6^b^	6.6 ± 1.1^b^	5.8 ± 1.5^a, b^	4.3 ± 0.9^b^	1.8 ± 0.2^a,b^

^a ^*P *< 0.05, treated DM patients vs. untreated DM patients;

^b ^*P *< 0.05, untreated DM patients vs. HC subjects

#### b. Analysis of apoptotic PMNLs

Apoptosis of PMNLs, higher in DM, decreased significantly after both antihyperglycemic treatments (Table 2).

### Systemic inflammation

#### a. Positive acute phase protein

Fibrinogen and CRP levels, higher in DM, decreased significantly after both antihyperglycemic treatments (Table 2).

#### b. Negative acute phase proteins

Albumin and transferrin levels were similar to HC and did not change following antihyperglycemic treatment (Table 2).

### Severity of diabetes and OS

**Figure 2:**
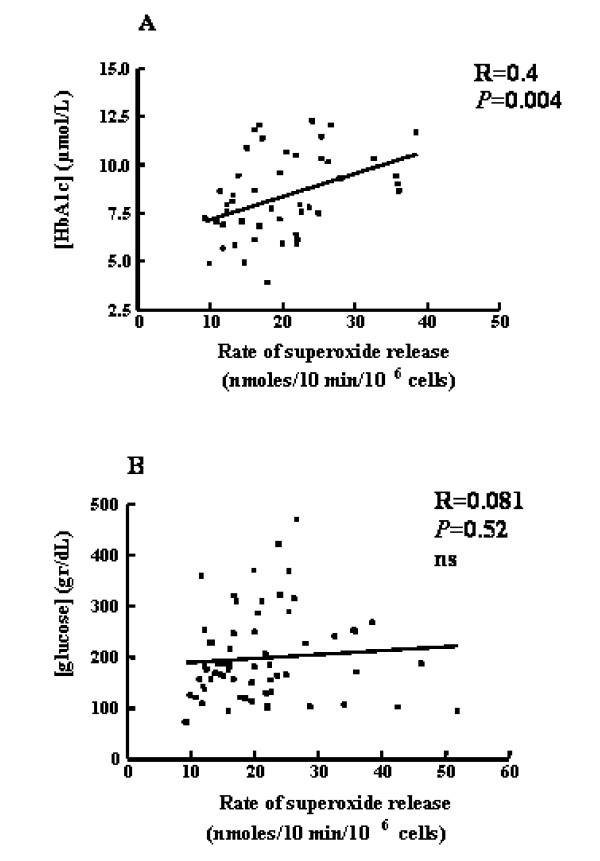
Correlation between the rates of superoxide release from separated PMA-stimulated PMNLs and either blood HbA1c (**A**) or blood glucose levels (**B**). Data refers to values from DM patients and HC subjects.

**Figure 3:**
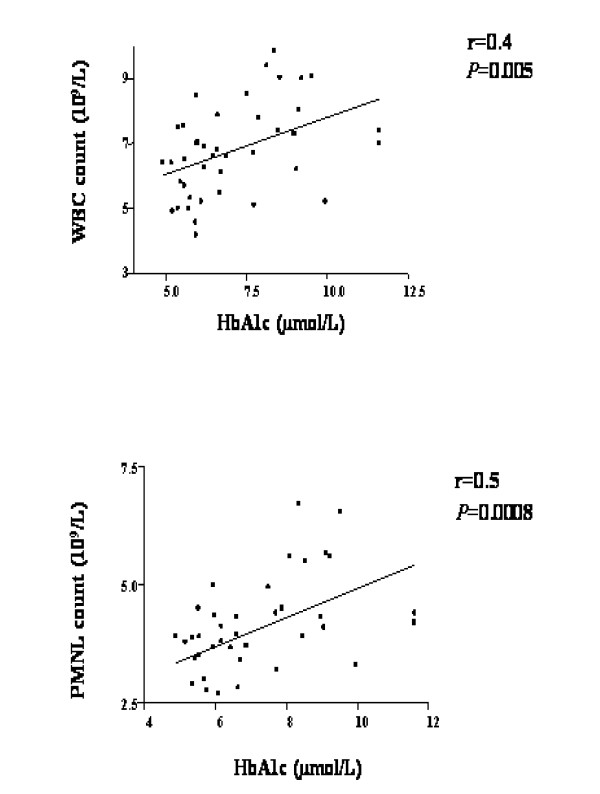
Correlation between blood HbA1c levels and either (**A**) WBC or PMNL (**B**) counts. Data refers to values from DM patients and HC subjects.

Blood HbA1c but not blood glucose levels were significantly positively correlated with the rates of superoxide release from PMA-stimulated PMNLs separated from all studied DM patients (R = 0.4, *P *= 0.004, Figure 2A; R = 0.081, *P *= 0.52, Figure 2B, respectively). Similar correlation was found with the second parameter of PMNL priming, namely CD11b (R = 0.4; *P *= 0.03). Similarly, Blood HbA1c levels were significantly positively correlated with WBC (R = 0.4, *P *= 0.005) or with PMNL counts (R = 0.5, *P *= 0.0008; Figure 3, respectively). These findings are indicative of relation between OS and the severity the disease, as reflected by HbA1c levels.

## Discussion

Our results show that type 2 diabetic patients are exposed to oxidative stress and chronic inflammation (partially because of the primed state of their PMNLs) before any clinical evidence of angiopathy exist [15]. When separated, PMA-stimulated diabetic PMNLs release superoxide significantly faster than normal control PMNLs and concomitantly with a reduced level of the plasma antioxidant GSH, predisposing these patients to OS [15]. Thirty untreated DM patients patients were included in this study: All the participants in this study were in age range of 30–65 year and nonsmokers. Patients underwent medical evaluation including opthalmoscopic examination, ECG and laboratory tests. The untreated DM patients were further enrolled in a prospective longitudinal study, being followed up for 4 and 8 weeks of either metformin (850 mg/day) or rosiglitazone (4 mg/day) treatment. A group of age- and gender-matched healthy controls (HC, n = 20) were included in the study.

Blood HbA1c and glucose levels significantly decreased following both treatment. Insulin levels, significantly higher in DM compared to HC, decreased following both antihyperglycemic treatment. Lipid profile (LDL-C, triglycerides) was also improved after two months following antihyperglycemic treatment as was described in literature [25]. PMNL priming parameters revealed different picture with each antihyperglycemic treatment; the metformin-treated patients showed significant increase in PMNL priming, as reflected by significant increase either in the rate of superoxide release from separated PMNLs or in CD11b levels on PMNLs. However, the rosiglitazone treatment inhibited significantly either the rate of superoxide release from separated PMNLs or in CD11b levels on PMNLs. It has to be emphasized that both groups of patients showed higher values of PMNL priming compared to those measured in HC subjects. ALP was higher in DM compared to HC and decreased during 2 months of both treatments. Along with its role as an hepatic enzyme, former studies have shown significantly higher blood level of this enzyme in hypertensive patients and rats, following degranulation from primed PMNLs [14]. The present study adds DM patients to this finding, and shows ameliorating results following both antihyperglycemic treatment. Counts of WBC and PMNLs, significantly higher in DM compared to HC, decreased only after rosiglitazone treatment, while metformin treatment caused a rise in these cells' count. The significant higher level of monocytes in DM patients, decreased significantly after both antihyperglycemic treatments. Albumin and transferrin levels were similar to HC and did not change following antihyperglycemic treatment. Other inflammatory markers like fibrinogen and CRP levels, higher in DM, decreased significantly after both antihyperglycemic treatments. Apoptosis of PMNLs, higher in DM, decreased significantly after both antihyperglycemic treatments. Blood HbA1c but not blood glucose levels were significantly positively correlated with the rates of superoxide release from PMA-stimulated PMNLs separated from all studied DM patients (R = 0.4, *P *= 0.004, R = 0.081, *P *= 0.52, respectively). Similar correlation was found with the second parameter of PMNL priming, namely CD11b (R = 0.4; *P *= 0.03). Similarly, Blood HbA1c levels were significantly positively correlated with WBC (R = 0.4, *P *= 0.005) or with PMNL counts (R = 0.5, *P *= 0.0008; respectively). These findings are indicative of relation between OS and the severity of the disease, as reflected by HbA1c levels. Likewise, multiple clinical trials show that reduction of HbA1c and blood glucose with the known antihyperglycemic treatments was optimal but all other inflammatory markers were worsen during the same treatment [21-26]. There is a great disagreement if tight control of glycaemia and decreasing HbA1c is sufficient to prevent the danger and risk for cardiovascular diseases and atherosclerosis.

## Conclusion

Our present research adds new facet in evaluating anti-hyperglycemic treatment in type 2 DM patients. Despite sufficient glycemic control achieved using both treatments, some PMNL-related parameters deteriorated. Thus, anti hyperglycemic treatment should be favored due to its combined anti-PMNL priming and anti-inflammatory effect, in addition to its anti-hyperglycemic characteristics, according to the correlation among these parameters. Such combined treatment may reduce morbidity and mortality common in DM patients [30,31]. Multiple other studies showed the beneficial effects of Thiazolidinediones (rosiglitazone) on cardiovascular disease in diabetes beyond improving blood glucose control [32-36]. There is a great need to complete this research with more time periods and sample to check the relation between the OS index and other inflammatory markers and the various anti hyperglycemic treatments in the reduction of mortality and morbidity.

## Abbreviations

DM: Diabetes mellitus; HbA_1c_: glycosylated hemoglobin A; HC: healthy control subjects; OS: oxidative stress; PMA: phorbol 12-myristate 13-acetate; PMNLs: polymorphonuclear leukocytes; ROS: reactive oxygen species; SOD: superoxide dismutase.
